# Supplementary material for: Redox Modification of PKA-Cα Differentially Affects Its Substrate Selection
Source: Life (Basel). 2023 Aug 26;13(9):1811. doi: 10.3390/life13091811 (PMC10532679; doi:10.3390/life13091811)
Supplement: Supplementary file 1 [file life-13-01811-s001.zip › life-2493158-supplementary.pdf]

Supplementary Information

Delva-Wiley et al.

A

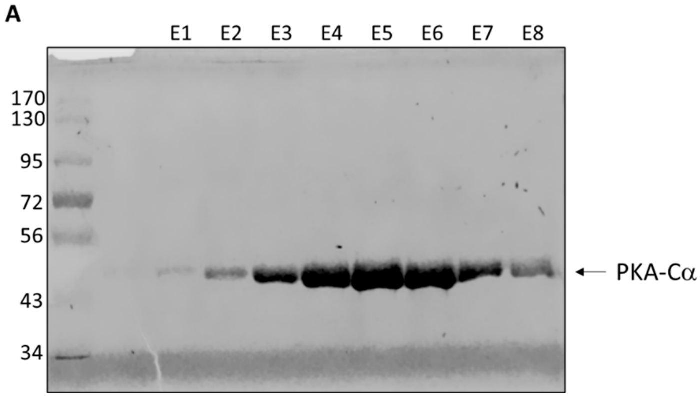

B

Protein Name	Process Step	Purity (%)	Conc (mg/mL)
WT PKA-Cα	Ni-NTA	60	1.03
	SP CEX	>95	3.23
PKA-Cα (C199A)	Ni-NTA	55	1.53
	SP CEX	>95	9.87
PKA-Cα (C199S)	Ni-NTA	65	1.23
	SP CEX	92	7.18

***Figure S1. Purification of PKA-Cα. A**. SDS-PAGE gel, stained with colloidal Coomassie-G250, following SP cation exchange (SP CEX) chromatography. The SP product exhibited a migration pattern consistent with His$_6$-PKA-Cα (arrow). **B.** Process purification summary for wild-type (WT) PKA-Cα, PKA-Cα (C199A), and PKA-Cα(199S).*

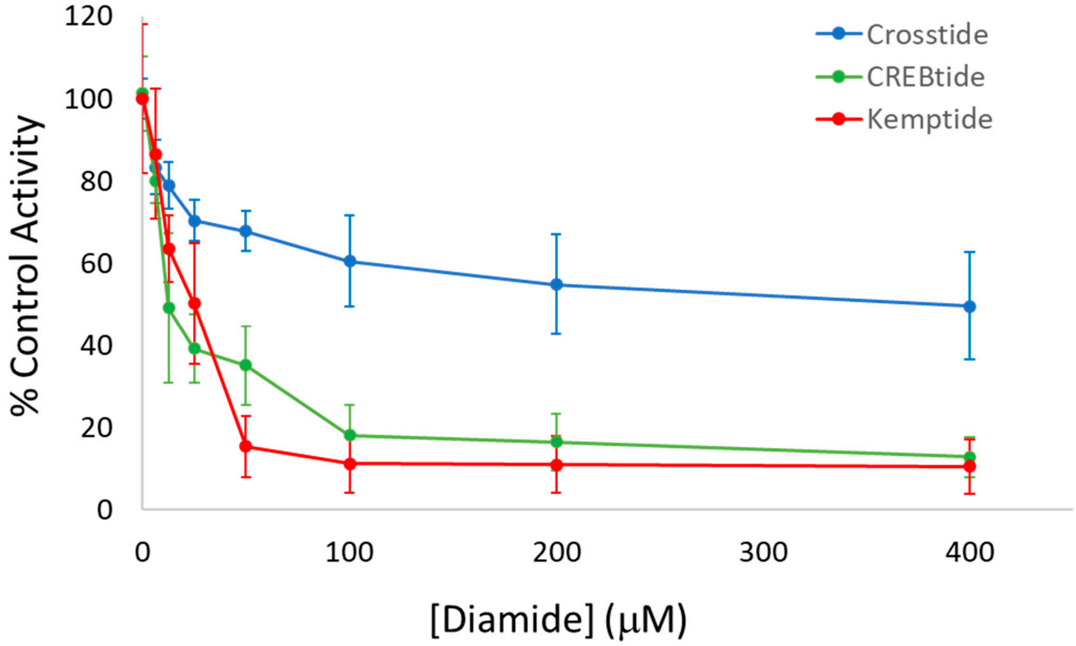

Figure S2. Impact of diamide-mediated oxidation on PKA-Cα toward different model substrates following an extended incubation with diamide. *Normalized relative activity of PKA-Cα toward Kemptide (red), CREBtide (green) and Crosstide (blue) following pre-treatment with the indicated concentrations of diamide for 20 minutes at room temperature.*

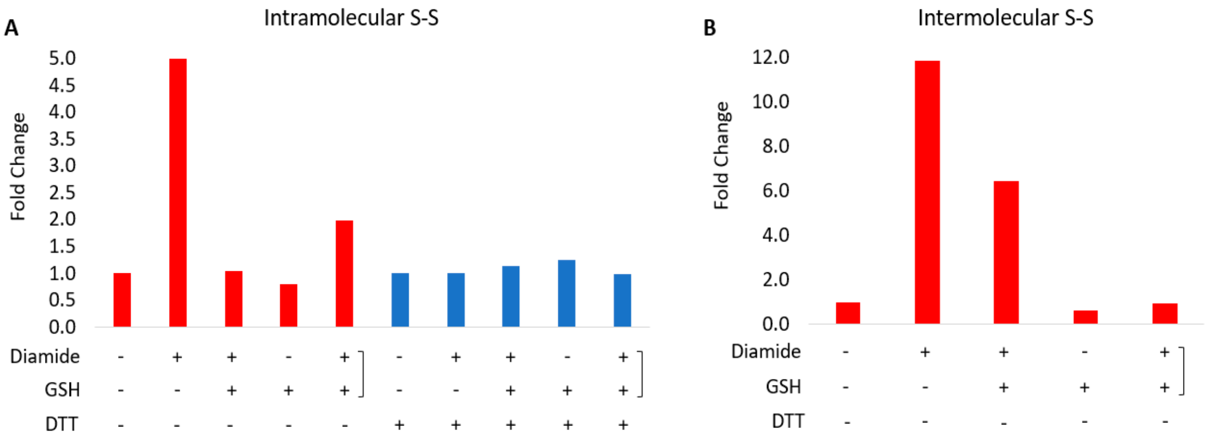

Figure S3. Quantitation of band intensities for intra- and intermolecular disulfide bonded species in non-reducing PAGE following treatment with diamide and/or glutathione. A. *Normalized intensities, expressed as fold change relative to the untreated control, of the faster migrating species in Figure 1D (corresponding to PKA-Cα that has formed an intramolecular disulfide bond) in the absence (red) and presence (blue) of reducing agent. DTT: dithiothreitol; GSH: reduced glutathione. Brackets in wells 5 and 10 indicate that diamide and GSH were added simultaneously.* ***B.*** *Normalized intensities, expressed as fold change relative to the untreated control, of the slower migrating species in Figure 1D (corresponding to PKA-Cα that has formed intermolecular disulfide bonds) in the absence of reducing agent. Abbreviations are as in* ***A****.*

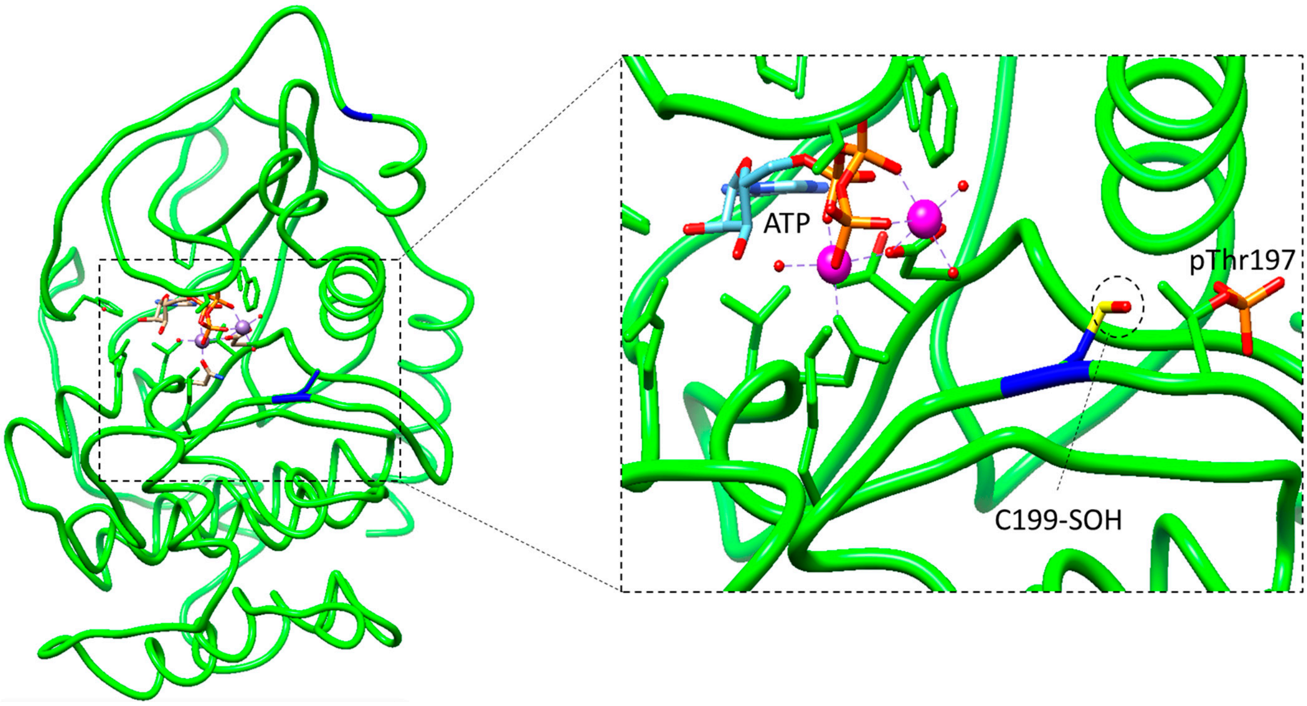

Figure S4. Sulfenylated C199 is well accommodated within PKA-Ca's active site. *The sulfhydryl group of C199 in PKA-Ca bound to ATP (PDB: 1ATP) was converted to sulfenic acid in silico using the "build" tool in Chimera-X. The modified structure was then energy minimized by molecular dynamics simulation by UCSF Chimera-X. Sulfenylated C199 (C199-SOH), phospho-T197 (pThr197) and ATP are labeled.*

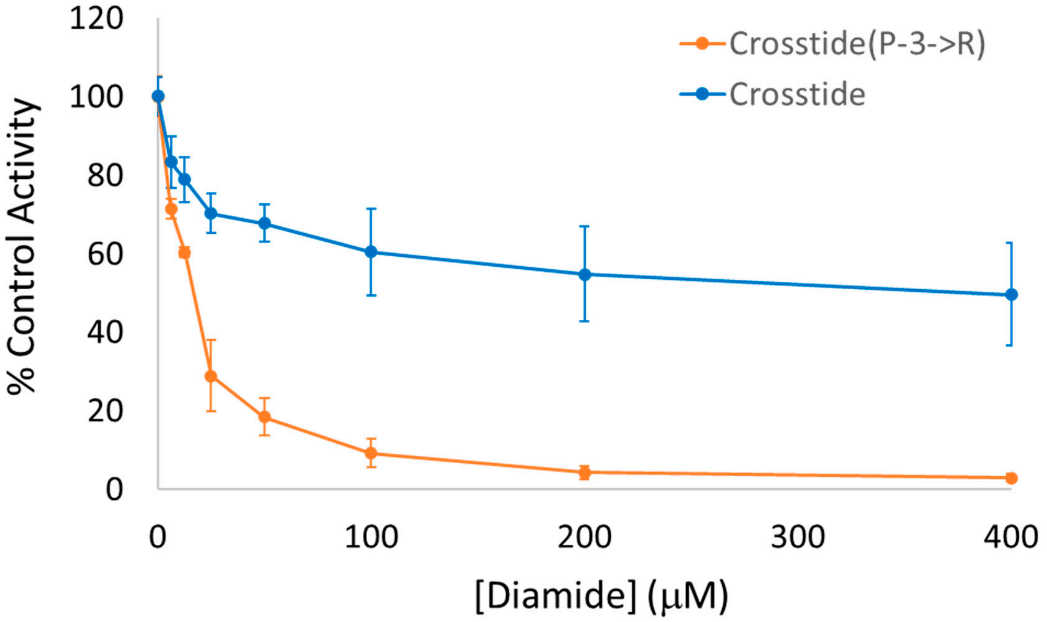

Figure S5. Impact of diamide-mediated oxidation on PKA-Cα toward Crosstide and Crosstide(P-3→R). *Normalized relative activity of PKA-Cα toward Crosstide (blue) and a Crosstide variant in which the Pro residue in the P-3 position has been mutated to Arg (Crosstide(P-3→R)) following pre-treatment with the indicated concentrations of diamide for 10 minutes at room temperature. Error bars represent standard error about the mean of three independent experiments run in duplicate.*

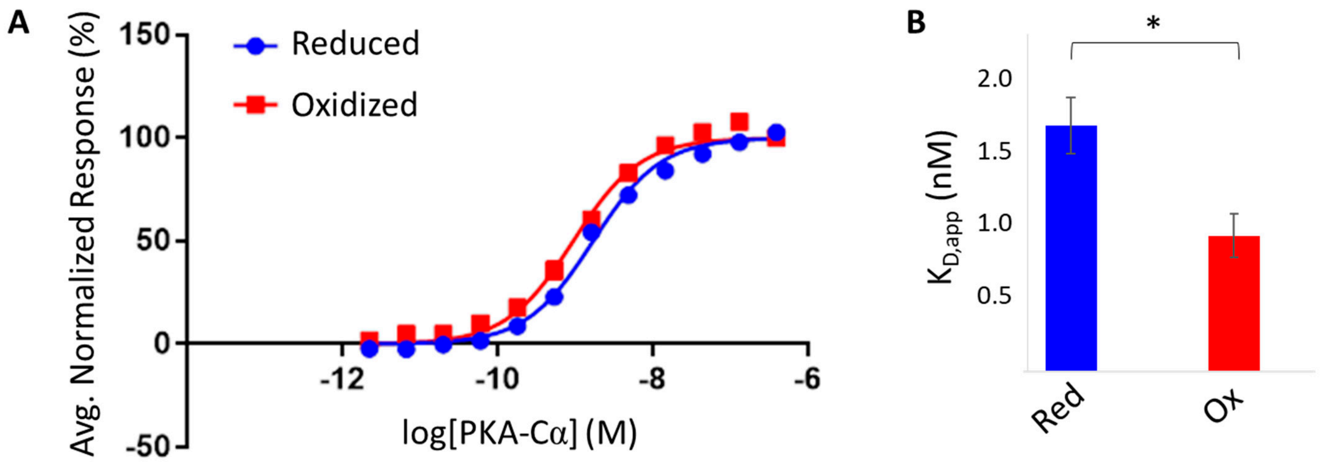

Figure S6. Fluorescence polarization assays to assess the impact of H_2O_2-dependent oxidation on PKA-Cα interactions with FAM-PKI(5-24). ***A.*** *Desalted PKA-Cα was treated with either dH_2O (blue circles) or a 20-fold molar excess of H_2O_2 (red squares) for 10 minutes at room temperature before scavenging excess H_2O_2 with catalase. The treated PKA-Cα was then incubated with FAM-labeled PKI(5-24) at the indicated concentrations at room temperature for 30 minutes before measuring fluorescence polarization of FAM-PKI(5-24). Error bars represent the standard error about the mean of 3 independent experiments conducted in duplicate.* ***B.*** *Average $K_{D,app}$ of PKA-Cα for PKI(5-24) following treatment with dH_2O (blue) or 5 µM H_2O_2 alone (red). Error bars represent SE about the mean of at least three independent experiments done in duplicate. *: $p < 0.05$.*

PRKACA	GYIQVTDFGFAKRV-----KGRTWTLCGTPEYLAPEIILS	213
PRKACB1	GYIQVTDFGFAKRV-----KGRTWTLCGTPEYLAPEIILS	260
PRKACG	GYLQVTDFGFAKRV-----KGRTWTLCGTPEYLAPEIILS	213
PRKCA	GHIKIADFGMCKEHMM-DG-VTTRTFCGTPDYIAPEIIAY	512
PRKCB1	GHIKIADFGMCKENIW-DG-VTTKTFCGTPDYIAPEIIAY	515
PRKCD	GHIKIADFGMCKENIF-GE-SRASTFCGTPDYIAPEILQG	522
PRKCH	GHIKITDFGMCKENVF-PG-TTTRTFCGTPDYIAPEILLG	529
PRKCI	GHIKLTDYGMCKEGLR-PG-DTTSTFCGTPNYIAPEILRG	427
PRKCQ	GHIKIADFGMCKENML-GD-AKTNTFCGTPNYIAPEILRG	553
PRKCZ1	GHIKLTDYGMCKEGLG-PG-DTTSTFCGTPNYIAPEILRG	425
PRKG1	GYAKLVDFGFAKKIGF---GKKTWTFCGTPEYVAPEIILN	532
PRKAA1	MNAKIADFGLSNM-MS-DG-EFLRTSCGSPNYAAPEVISG	189
PRKAA2	MNAKIADFGLSNM-MS-DG-EFLRTSCGSPNYAAPEVISG	187
RPS6KA1	GHIKLTDFGLSKEAID-HE-KKAYSFCGTVEYMAPEVVNR	236
RPS6KA2A	GHIKITDFGLSKEAID-HD-KRAYSFCGTIEYMAPEVVNR	233
RPS6KA3	GHIKLTDFGLSKESID-HE-KKAYSFCGTVEYMAPEVVNR	242
RPS6KA4A	GHIVLTDFGLSKEFLT-EEKERTFSFCGTIEYMAPEIIRS	211
RPS6KA5A	GHVVLTDFGLSKEFVA-DETERAYSFCGTIEYMAPDIVRG	227
RPS6KA6	GHIKLTDFGLSKESVD-QE-KKAYSFCGTVEYMAPEVVNR	247
AKT1	GHIKITDFGLCKEGIK-DG-ATMKTFCGTPEYLAPEVLED	323
AKT2	GHIKITDFGLCKEGIS-DG-ATMKTFCGTPEYLAPEVLED	324
AKT3	GHIKITDFGLCKEGIT-DA-ATMKTFCGTPEYLAPEVLED	320
SGK1	GHIVLTDFGLCKENIE-HN-STTSTFCGTPEYLAPEVLHK	271
SGK2A	GHVVLTDFGLCKEGVE-PE-DTTSTFCGTPEYLAPEVLRK	208
SGK3	GHVVLTDFGLCKEGIA-IS-DTTTTFCGTPEYLAPEVIRK	335
CAMK1A	SKIMISDFGLSKM-ED-PG-SVLSTACGTPGYVAPEVLAQ	192
CAMK1D	SKIMISDFGLSKM-EG-KG-DVMSTACGTPGYVAPEVLAQ	195

Figure S7. Conservation of redox sensitive cysteines among AGC kinase family members. *C199 in the P+1 loop of PKA-Cα (PRKACA) is conserved among many AGC kinases. The conserved Cys is highlighted in red. Those sites that are known to be oxidized are underlined.*
